# Analysis of Commercial Proanthocyanidins. Part 6: Sulfitation of Flavan-3-Ols Catechin and Epicatechin, and Procyanidin B-3

**DOI:** 10.3390/molecules25214980

**Published:** 2020-10-28

**Authors:** Anwar E.M. Noreljaleel, Anke Wilhelm, Susan L. Bonnet

**Affiliations:** Department of Chemistry, University of Free State, Bloemfontein 9301, South Africa; anwarelbushra@yahoo.ca (A.E.M.N.); WilhelmA@ufs.ac.za (A.W.)

**Keywords:** sulfitation, tannins, *Acacia mearnsii* de Wild., flavan-3-ol, C-2 sulfonation, PD4 calculations, procyanidin B-3, C-4 sulfonation

## Abstract

Proanthocyanidins (PACs) are natural plant-derived polymers consisting of flavan-3-ol monomers. Quebracho (*Schinopsis lorentzii* and *balansae*) heartwood and mimosa (*Acacia mearnsii*) bark extracts are the major industrial sources of PACs. These commercial extracts are often sulfited to reduce their viscosity and increase their solubility in water. The chemical process of sulfitation is still poorly understood regarding stereochemical influences during the reaction and during the cleavage of the interflavanyl bond of oligomers. To gain a better understanding of sulfitation, two diastereomeric flavan-3-ol monomers were sulfited under industrial conditions, and procyanidin B-3 (catechin-4α→8-catechin) were sulfited to investigate interflavanyl bond cleavage with sulfitation at C-4. Treatment of diastereomeric flavan-3-ols 2*R*,3*S*-catechin and 2*R*,3*R*-epicatechin with NaHSO_3_ at 100 °C in aqueous medium afforded the enantiomeric (1*R*,2*S*)- and (1*S*,2*R*)-1-(3,4-dihydroxyphenyl)-2-hydroxy-3-(2,4,6-trihydroxyphenyl)propane-1-sulfonic acid, respectively. Utilizing computational NMR PD4 calculations it was determined that the direction of stereoselective nucleophilic attack is controlled by the C-3 configuration of the flavan-3-ols catechin and epicatechin. Sulfitation of the catechin-4α→8-catechin dimer **7** (procyanidin B-3) under the same conditions led to the cleavage of the interflavanyl bond yielding the C-4 sulfonic acid substituted catechin momomer. From the heterocyclic ring coupling constants it was determined that nucleophilic attack occurs from the β-face of the dimer leading to the 2,3-*trans*-3,4-*cis* isomer as product.

## 1. Introduction

**Figure 1:**
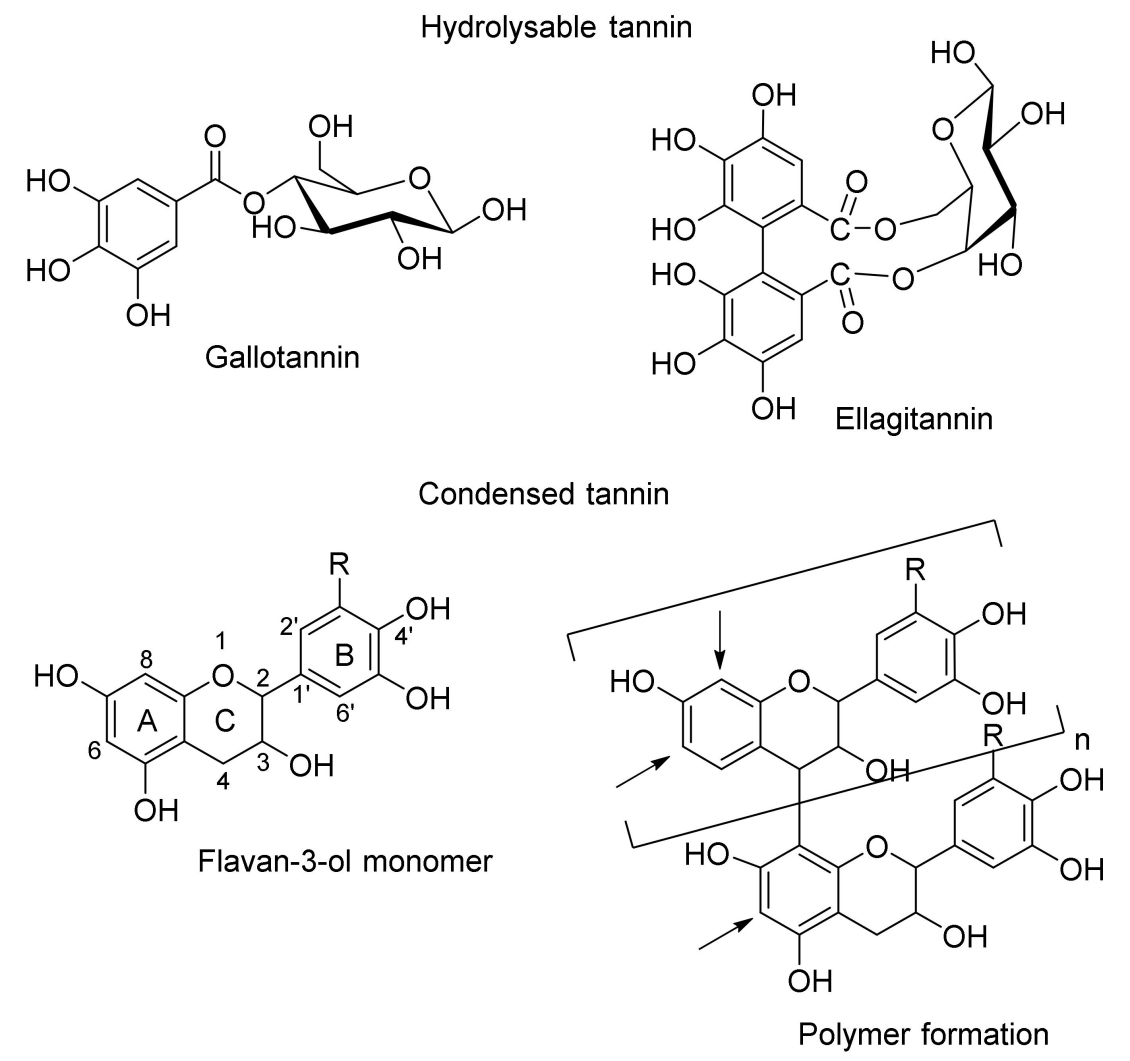
Basic structures of hydrolysable and condensed tannins.

The tanning of animal skins or hides into durable leather has been practiced since antiquity, and the original method used was vegetable pit tanning [1]. Vegetable material containing tannins reacts irreversibly with the protein constituents of hides to yield soft leather, which is resistant to microbial degradation, water, heat and abrasion [2]. Tannins are secondary metabolites found in higher plants and mainly consist of hydrolysable tannins (polyesters of gallic or hexahydroxydiphenic acid and D-glucose) and proanthocyanidins (PACs), also known as condensed tannins (complex oligomers of flavan-3-ol monomers) (Figure 1). The four major trees from which tanning material is extracted are mimosa (*Acacia mearnsii* bark) and quebracho (*Schinopsis lorenzii* and *S. balansae*, heartwood) for condensed tannins [3,4] and chestnut (*Castanea sativa* and *C. dentate*, wood) [5] and tara (*Caesalpinia spinosa*, pods) for hydrolysable tannins [6].

Hydrogen bond formation between the hydroxy groups of the polyphenolic PACs and proteins forms the basis for many of the tannin extract applications. In plants the formation of indigestible PAC-protein complexes gives rise to the anti-feeding effect and protects the plants from herbivores [7]. Industrially, PAC extracts are still widely used in the leather industry [8], the adhesive industry [9], as additives to animal feed [10], the wine industry [11], as flocculants [12,13,14], and as mud-drilling additives [13]. Pharmacological properties include anti-oxidant activity [15,16,17], Alzheimer’s disease inhibition [18], anti-cancer activity [19,20], anti-diabetic [21,22], anti-microbial [23,24] and anti-cardiovascular diseases [25,26].

Tannins have been chemically modified for the development of new biobased polymers to enhance industrial applications. The main sites on the condensed tannin structure for modifications are the phenolic hydroxy groups (etherification and esterification [27,28]), opening of the heterocyclic ring with substitution at C-2 (e.g., sulfitation [29]), electrophilic aromatic substitution on mainly the more reactive A-ring at positions 6 and 8 (e.g., bromination [30,31]), condensation reactions [32], reactions with aldehydes leading to polymerization via methylene bridges [33] and methylamination via the Mannich reaction [30,34,35].

**Figure 2:**
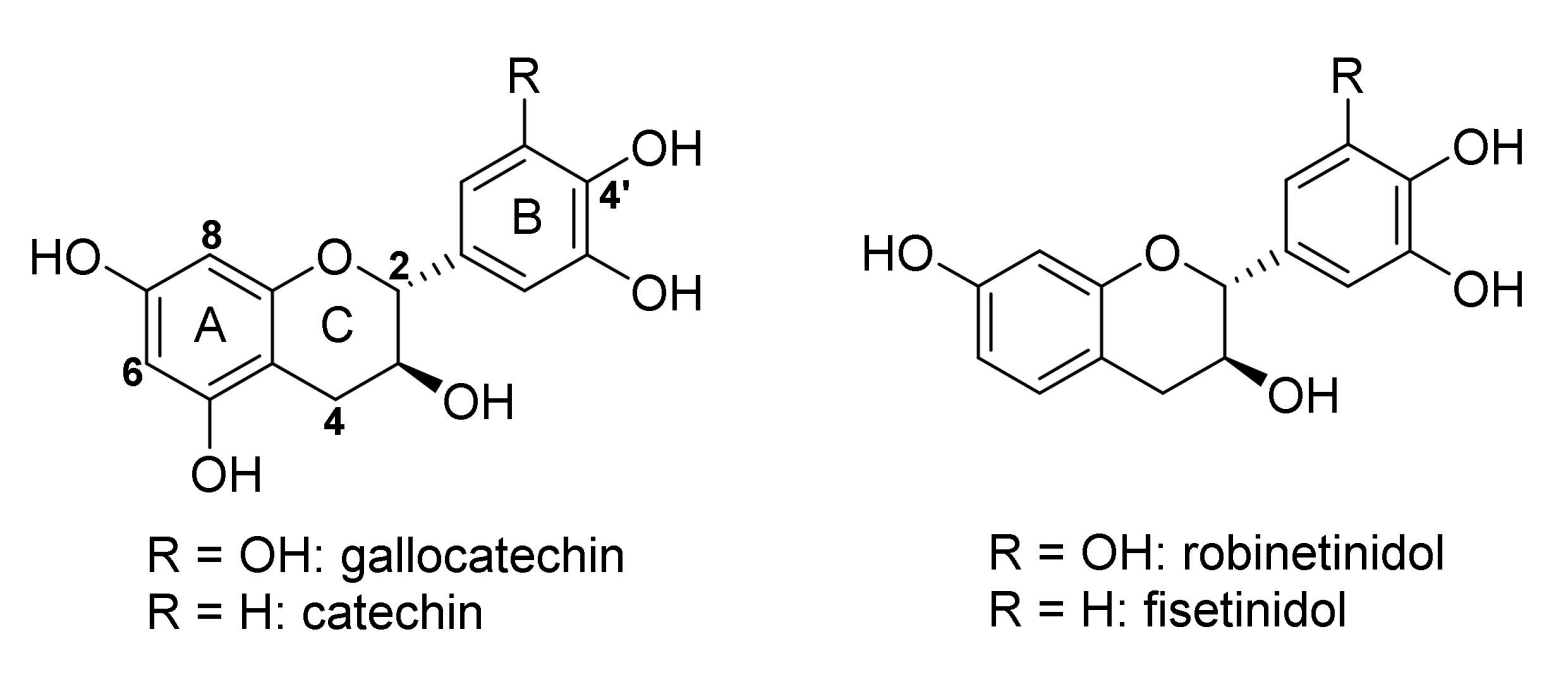
Flavan-3-ol monomers isolated from *Acacia mearnsii* De Wild. bark.

The sulfitation of condensed tannins is one of the oldest modification reactions and changes the physical and chemical properties of PAC extracts. The reaction greatly improves the water solubility of PACs and also reduces the viscosity of PAC extracts [30]. Furthermore, the moisture retention of tannin adhesives is enhanced and consequently the adhesive film dries more gradually [29]. During sulfitation the heterocylic C-ring of the flavan-3-ol monomer building block is cleaved, affording sulfonic acid derivatives [29,36], and secondly, the interflavanyl bond between two flavan-3-ol units can be cleaved. Extensive studies on the composition of the unsulfited and sulfited PAC extracts from quebracho heartwood and black wattle bark via low and high resolution electron spray ionization mass spectrometry (LR-ESIMS and HR-ESIMS) supported these findings [37,38,39,40]. They concluded that the flavan-3-ol building blocks of quebracho heartwood PACs were catechin as starter unit and fisetinidol as extender unit [37], while PACs in black wattle bark extract comprise catechin and gallocatechin starter units, and fisetinidol and robinetinidol extender units [39] (Figure 2).

**Figure 3:**
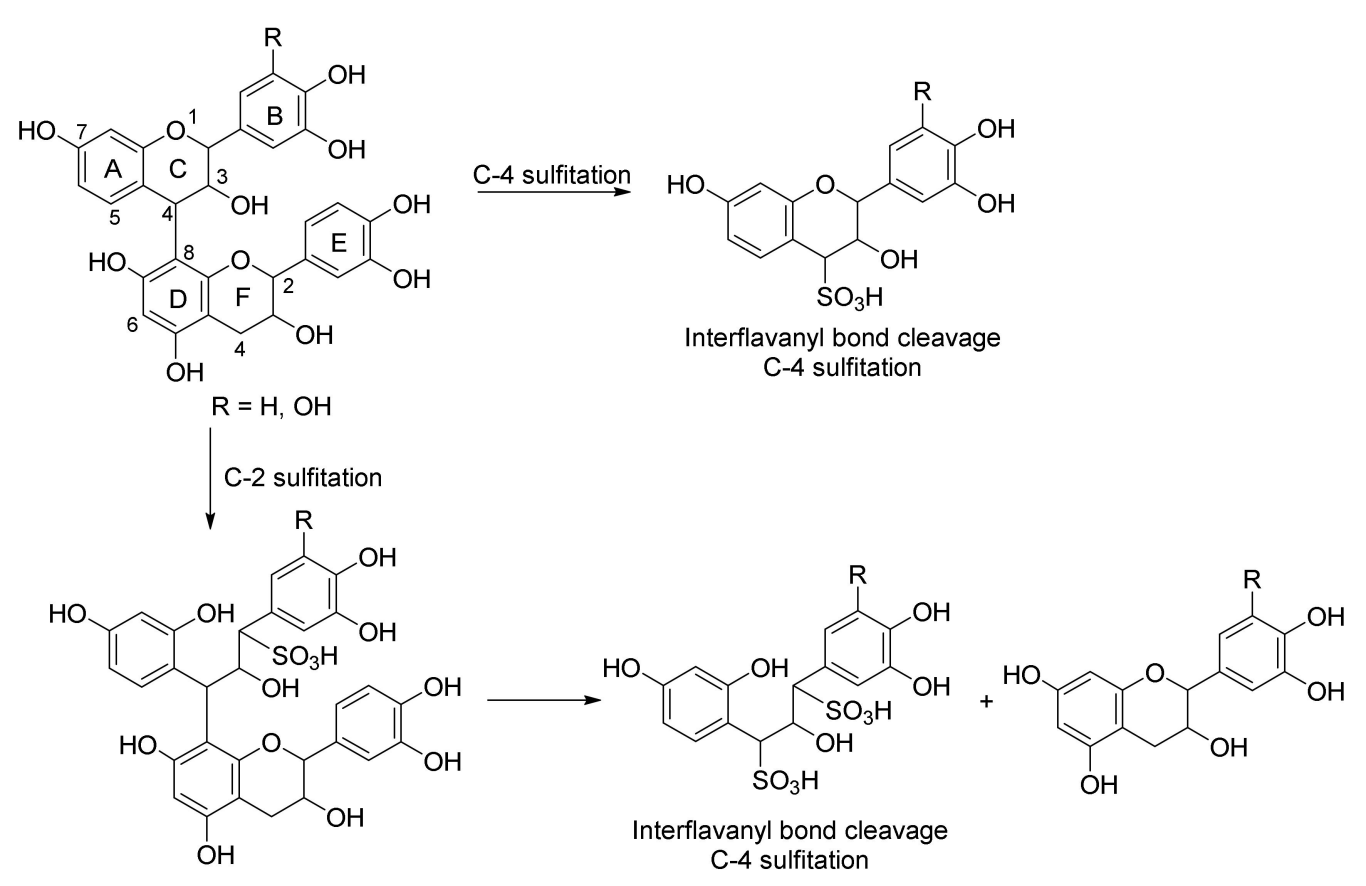
Sulfitation of a PAC dimer affords C-2 and C-4 (interflavanyl bond fission) sulfonated products.

Sulfitation of the quebracho PACs thus transforms the hot-water-soluble extract into a cold-water-soluble extract, since the introduction of sulfonic acid moieties increases the polarity of the molecules [38]. They also reported that no sulfitation at the C-4 positions of the starter units catechin and gallocatechin was observed, but that interflavanyl bond cleavage affords sulfitation at C-4 of the extender units, shortening chain lengths [37,38,39,40]. They thus deduced that C-4 sulfitation only occurs on the extender units (Figure 3).

However, even though sulfitation of tannin structures is a well-known reaction, the chemical process of sulfitation is still poorly understood regarding stereochemical influences during the reaction and the cleavage of the interflavanyl bond of oligomers. To gain a better understanding of sulfitation, two diastereomeric flavan-3-ol monomers were sulfited under industrial conditions, and procyanidin B-3 (catechin-4α→8-catechin) were sulfited to investigate interflavanyl bond cleavage.

## *2.* Results and Discussion

### 2.1. Sulfitation of Diastereomeric 2R,3S-Catechin and 2R,3R-Epicatechin

**Scheme 1:**
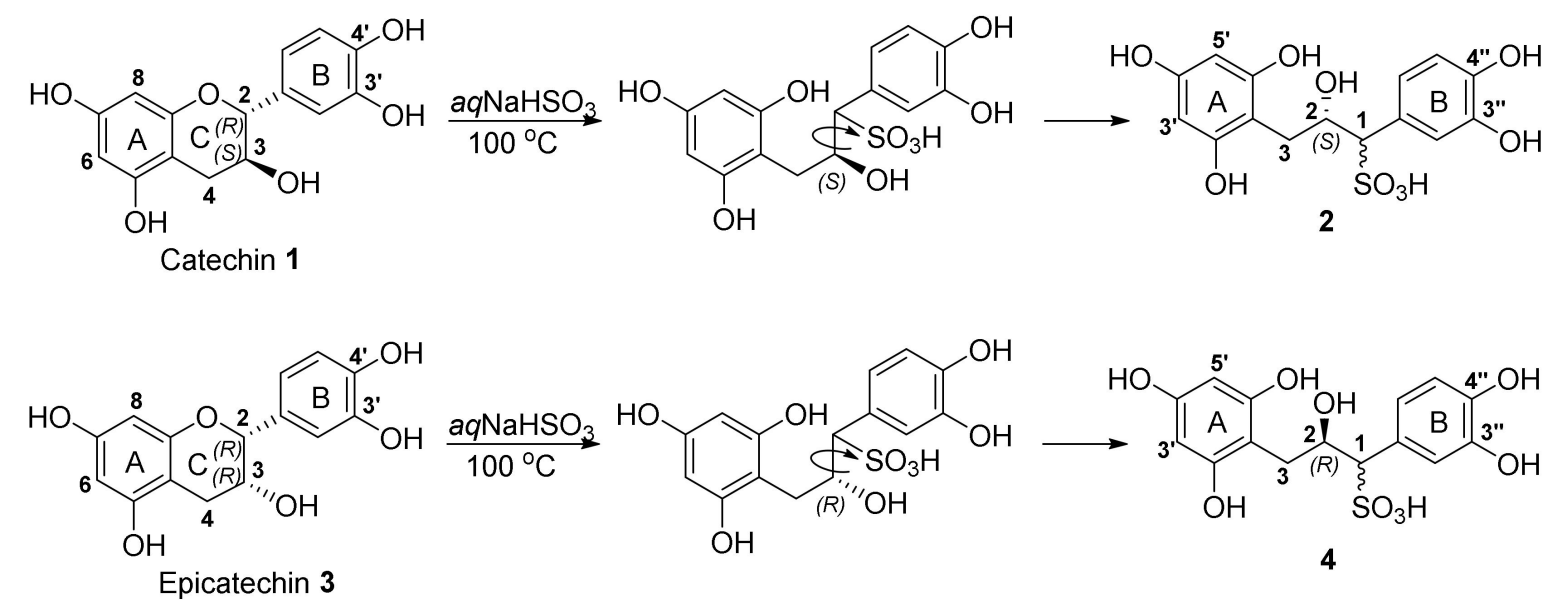
Sulfitation of 2*R*,3*S*-catechin 1 and 2*R*,3*R*-epicatechin 3.

Treatment of diastereomeric flavan-3-ols 2*R*,3*S*-catechin (**1**) and 2*R*,3*R*-epicatechin (**3**), respectively, with NaHSO_3_ at 100 °C in aqueous medium afforded only one major ring-opened product in each reaction, **2** and **4**, respectively, with a sulfonic acid group at C-1 (Scheme 1).

During structure elucidation it was observed that products **2** and **4** had identical ^1^H and ^13^C NMR spectra, but opposite Cotton effect electron circular dichroism (ECD) spectra, indicating an enantiomeric relationship between **2** and **4**. The aromatic region of the ^1^H NMR spectra of enantiomers **2** and **4** show one two-proton singlet at δ_H_ 5.87 and 5.88, respectively, corresponding to the equivalent H-3′ and H-5′ of the free-rotating phloroglucinol A-ring, and one ABX system corresponding to the B-ring at δ_H_ 6.75 and 6.76 (d, H-5′′) and 6.78 and 6.79 (dd, H-6′′), respectively, and 6.95 (d, H-2′′) for both **2** and **4**. In the aliphatic region, H-1 is observed at δ_H_ 3.81 as a triplet of doublets, H-2, situated on the oxygenated C-2, at δ_H_ 4.53, and H-3eq and H-3ax is observed as two doublet of doublets at δ_H_ 2.79 and 2.45, respectively.

**Scheme 2:**
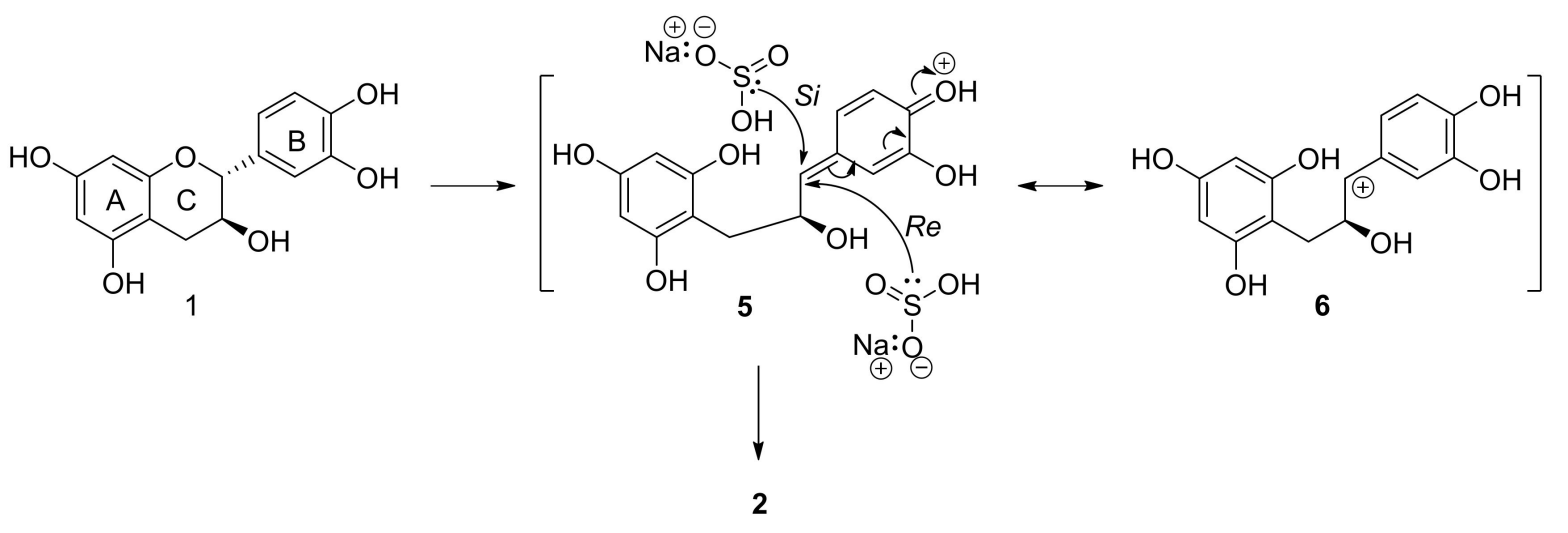
Proposed mechanism of the sulfitation of catechin **1**.

Owing to free rotation about aliphatic bonds, the absolute configuration at C-1 of **2** and **4** cannot be determined via aliphatic coupling constants in the ^1^H NMR spectra. However, the enantiomeric relationship implies that the absolute configuration at C-1 of the products are determined by the absolute configuration at the C-3 chiral center of the starting materials. It has been postulated that cleavage of the heterocyclic C-ring of **1** and **3** can result in two possible tautomeric intermediates [41]: either the *para*-quinone methide of the B-ring (**5**), or the *para*-hydroxy stabilized benzylic cation (**6**) (Scheme 2). They further reported that the hydroxy-group at C-4′ is essential for substitution at C-2 of the starting materials. Both tautomeric intermediates **5** and **6** are planar, due to sp^2^-hybridization of C-1, and nucleophilic attack can thus occur either on the *Si*-face or the *Re*-face of the intermediates (Scheme 2).

**Figure 4:**
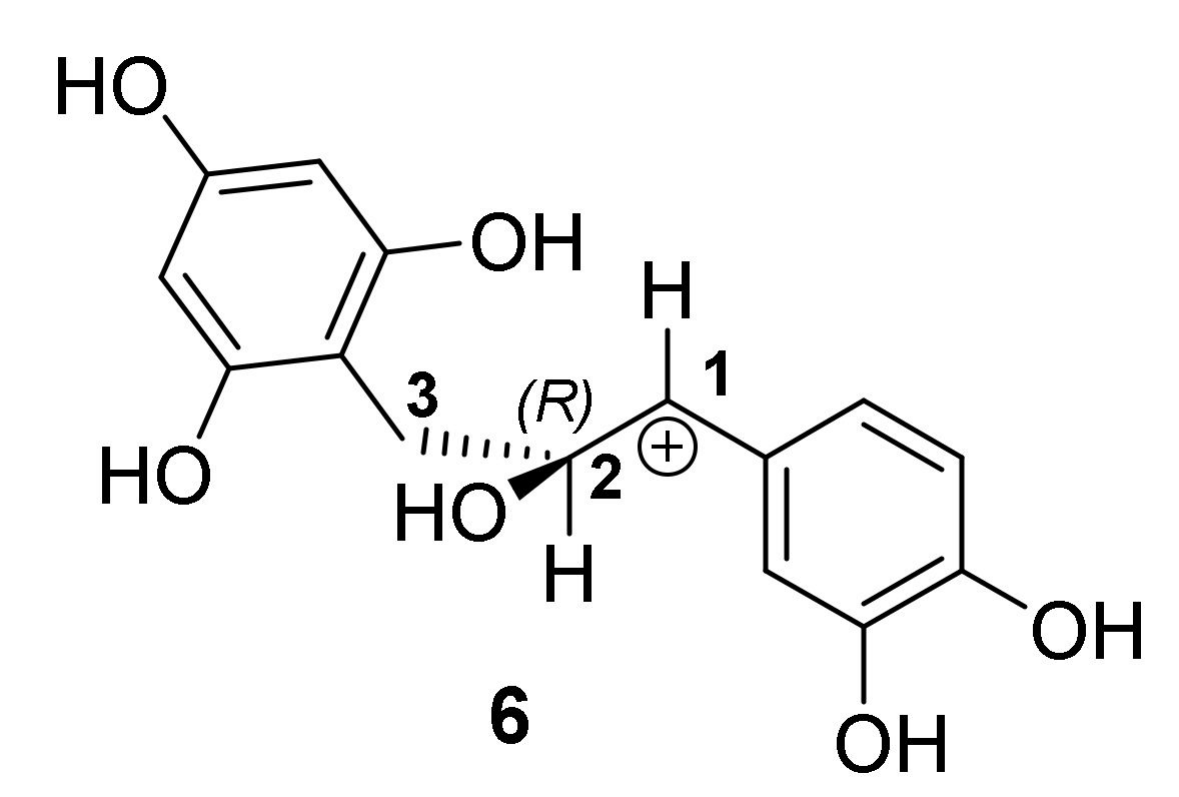
Most stable conformation of the benzylic carbocation intermediate (**6**) with the α-H in the 1,3-allylic strain position.

The absolute configuration at C-1 of the sulfonic acid adduct depends on the face of attack at the planar C-1 center in the tautomeric intermediates. Attack on the *Si*-face will afford 1*S* absolute configuration at C-1, while *Re*-face attack will give rise to 1*R* absolute configuration. Stereoselective attack can be directed by either the benzyl group or the hydroxy group at C-2, dependent on the preferred conformation of the aliphatic system. Bach and coworkers postulated that α-chiral secondary and tertiary benzylic carbocations have a preferred conformation where the α-hydrogen atom occupies the 1,3-allylic strain position (Figure 4) [42].

Diastereotopic faces of carbocations are differentiated by the functional groups at the α-carbon.

**Scheme 3:**
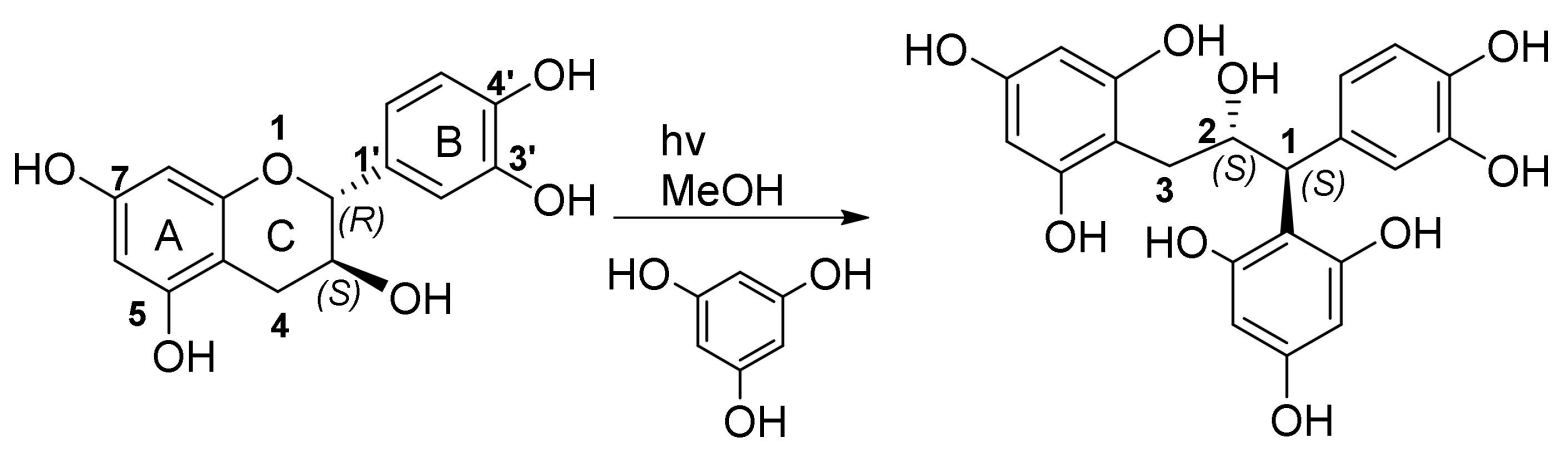
Photolysis of catechin in the presence of phloroglucinol.

In the case of intermediate **6**, this would imply that the bulkier benzyl group at C-2 and not the less bulky hydroxy group at C-2 will direct attack to the *Si*-face leading to the 1*S*,2*S* diastereomer. However, Wilhelm and co-workers [41] cleaved the heterocyclic ring of flavan-3-ols (catechin and epicatechin, respectively) via photolytic fission of the ether bond. Trapping of the intermediates with phloroglucinol afforded phloroglucinol-grafted derivatives, with identical NMR spectra indicating formation of enantiomers. In this case the C-2 absolute configuration was established via theoretical calculation of the electronic circular dichroism (ECD) spectra. The results indicated that the hydroxy group at C-3 directed attack to the *Re*-face leading to the 1*S*,2*S* diastereomer (Scheme 3).

In order to unequivocally determine the absolute configuration at C-1 of products **2** and **4**, gauge-invariant atomic orbital (GIAO) NMR shift calculation and DP4 (isomer probability parameter) analysis were performed.

### 2.2. GIAO NMR Shift Calculation and DP4 Analysis

Computational NMR prediction has become a common tool to calculate theoretical NMR shifts [43,44]. Smith and Goodman used gauge-invariant atomic orbital (GIAO) NMR shift calculations to determine stereochemistry in several pairs of diastereomers [45]. They subsequently applied GIAO NMR shift calculations to assign stereochemistry with quantifiable confidence when only one set of experimental data was available, formulating and applying the DP4 parameter, which aids in assigning structure and stereochemistry by comparing experimental and calculated NMR spectra [46]. The only requirements for this process are the experimentally defined ^1^H and ^13^C NMR chemical shifts. It involves the calculation of the shifts for the candidate structures (employing a Boltzmann weighted average of the shifts calculated for all low-energy conformers), followed by DP4 comparison to the experimental data to decide which gives the best match. The program also gives a measure of its confidence in its conclusion.

**Figure 5:**
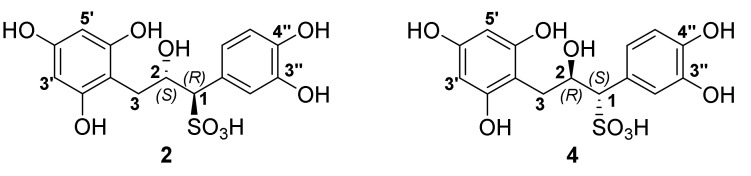
Absolute configurations of sulfited catechin (**2**) and sulfited epicatechin (**4**).

Thus, the calculated and experimental NMR data depicted in Table S1 and Table S2 were determined via GIAO NMR shift calculation and DP4 analysis of our proposed structures **2** and **4**, and the absolute configurations were indicated to be 1*R*,2*S* for **2** and 1*S*2*R* for **4** with 100% confidence (Figure 5 and Table 1).

Thus, stereoselective attack leads to the formation of enantiomeric products when catechin and epicatechin are sulfonated. The direction of nucleophilic attack is controlled by the C-3 configuration of the flavan-3-ols **1** and **3**. In the case of the catechin intermediate **5**/**6**, *Re*-face attack afforded (1*R*,2*S*)-1-(3,4-dihydroxyphenyl)-2-hydroxy-3-(2,4,6-trihydroxyphenyl)propane-1-sulfonic acid (**2**), and for epicatchin *Si*-face attack yielded (1*S*,2*R*)-1-(3,4-dihydroxyphenyl)-2-hydroxy-3-(2,4,6-trihydroxyphenyl)propane-1-sulfonic acid (**4**).

### 2.3. Sulfitation of Procyanidin B-3

**Scheme 4:**
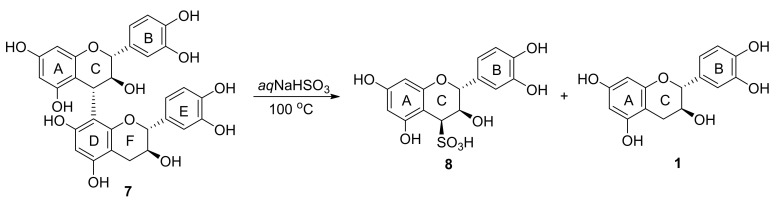
Sulfitation of procyanidin-B3.

Sulfitation of the catechin-4α→8-catechin dimer **7** (procyanidin B-3) under the same conditions led to the cleavage of the interflavanyl bond yielding the C-4 sulfonic acid substituted monomer **8** and **1** (Scheme 4).

The aromatic region of the ^1^H NMR spectrum indicated two doublets corresponding to the A-ring protons at δ_H_ 5.95 (H-6) and 6.04 (H-8) and an aromatic ABX system at δ_H_ 6.96 (H-2ʹ, d), 6.81 (H-5ʹ, d) and 6.83 (H-6ʹ, dd). The heterocyclic ring protons resonated at δ_H_ 5.53 (H-2, d), δ_H_ 4.13 (H-3, dd) and δ_H_ 4.52 (H-4, d). Van der Westhuizen et al. used the ^1^H NMR coupling constants of the C-ring resonances to distinguish between the diastereoisomers of 4-arylflavan-3-ols [47]. The characteristic coupling constants for 4-arylflavan-3-ols with a 2,3-*trans*-3,4-*cis* relative stereochemistry are *J*_2,3_ = 8–10 Hz and *J*_3,4_ = 5.0-6.5 Hz. Extrapolating to our 4-substituted flavan-3-ols, the coupling constants of *J*_2,3_ = 10.3 Hz and *J*_3,4_ = 5.1 Hz for **11** indicates a 3,4-*cis* relative stereochemistry, thus the absolute configuration at C-4 is 4*S* (Figure 1). This indicates that nucleophilic attack occurs selectively from the β-face of the heterocyclic ring, as expected, since the large C-4 substituent is blocking the α-face of the dimer. The cleavage of the interflavanyl bond in condensed tannin extracts leads to smaller oligomers that may be beneficial for leather tanning, since the preferable chain length for leather bonding is three to four monomers.

## 3. Conclusions

Treatment of the two diastereomeric flavan-3-ol monomers catechin and epicatechin, with NaHSO_3_ at 100 °C in aqueous medium, yielded sulfited enantiomeric products, which confirmed our previous findings [40] that sulfonic acid moieties (-SO_3_H) are stereoselectively introduced at C-2, with cleavage of the ether bond of the heterocyclic C-ring. It was demonstrated, via GIAO NMR shift calculations and DP4 analysis, that the stereochemistry at C-1 of the formed sulfited products is controlled by the C-3 configuration of the starting flavan-3-ols. Sulfitation of the catechin-4α→8-catechin dimer (procyanidin B-3) under the same conditions led to the cleavage of the interflavanyl bond yielding the 2,3-*trans*-3,4-*cis* C-4 sulfonic acid substituted monomer, exclusively. This finding substantiates our previous results [40] that cleavage of the interflavanyl bond leads to shorter chain lengths that contribute to the reduced viscosity, improved solubility and better raw skin penetration of sulfited wattle bark extract.

## 4. Materials and Methods

Chemicals purchased from commercial vendors were reagent/analytical grade and were used without purification. Catechin, epicatechin and sodium hydrogen sulfite were purchased from Sigma-Aldrich (Johannesburg, South Africa). Procyanidin B-3 was synthesized in-house and purity determined via HPLC. Solvents methanol, hexane and ethyl acetate were purchased from Merck (Johannesburg, South Africa).

A 600 MHz Bruker Avance II NMR spectrometer with a ^13^C and ^1^H 5 mm DUAL ^13^C-^1^H/D probe with z-gradients operating at 25 °C was used to record all NMR spectra at a ^1^H frequency of 600.28 MHz and a ^13^C frequency of 150.95 MHz. The instrument was manufactured by Bruker Biospin AG at Fällanden, Switzerland. The ^1^H NMR, COSY, HMBC, HSQC, ^13^C and APT experiments were performed in methanol-*d_4_*, (δ_H_ = 4.87 and 3.31; δ_C_ = 49.2) with tetramethylsilane (TMS) as internal standard. Chemical shifts were expressed as parts per million (ppm) on the delta (δ) scale and coupling constants (*J*) are accurate to 0.01 Hz.

Thin-layer chromatography (TLC) was performed on Merck aluminium sheets (silica gel 60 F_254_, 0.25 mm). Reactions were monitored using TLC on silica gel, with detection by UV light (254 nm). Thin-layer chromatograms were sprayed with a 2% (*v*/*v*) solution of formaldehyde (40% solution in water) in concentrated sulphuric acid and subsequently heated to 110 °C to effect maximum development of colour.

All conformational searches were conducted employing the Macromodel (Version 9.9, Schrodinger LLC., New York, NY, USA) program with “Mixed torsional/LowMode sampling” in the MMFF force field. The searches were performed in the gas phase with a 50 kJ/mol energy window limit and 10,000 maximum number of steps to fully explore all low-energy conformers. All minimization processes were carried out utilizing the Polak-Ribiere conjugate gradient (PRCG) method, 10,000 maximum iterations and a 0.001 convergence threshold. The GIAO shielding constants of all conformers within 10 kJ/mol of the global minimum were calculated utilizing a Gaussian 09 package (Gaussian Inc., Wallingford, CT, USA) at the B3LYP/6-31G(d,p) level in the gas phase. The calculation of DP4 probability was facilitated using an applet available at “http://www-jmg.ch.cam.ac.uk/tools/nmr/DP4/”.

General procedure for sulfitation:

The flavan-3-ol (or dimer) (100 mg), excess sodium hydrogen sulfite (100 mg) and water (4 mL) were sealed in a glass tube and heated at 100 °C for 8 h. The reaction mixture was cooled down, extracted with ethyl acetate (2 × 20 mL) to remove unreacted catechin and the water layer lyophilized. The crude was subsequently dissolved in methanol, the unreacted NaHSO_3_ filtered off and the solvent removed under vacuum to yield the amorphous product.

NMR data of all the products are available in the Supplementary Materials section, Table S1–S3.

## Figures and Tables

**Table 1:** DP4 probability results for products **2** and **4**.

Probability	Sulfited Catechin (2)		Sulfited Epicatechin (4)	
	1*R*2*S*	1*S*2*S*	1*R*2*R*	1*S*2*R*
^1^H NMR	99.7%	0.3%	2.3%	97.7%
^13^C NMR	96.1%	3.9%	38.9%	61.1%
Combined	100.0%	0%	0%	100.0%

## Supplementary Materials

The following are available online at https://www.mdpi.com/1420-3049/25/21/4980/s1, Table S1: Experimental and calculated ^1^H and ^13^C NMR shifts for compound **2**, Table S2: Experimental and calculated ^1^H and ^13^C NMR shifts for compound **4**, Table S3: ^1^H (600 MHz, MeOD, 20 °C) and ^13^C NMR data (150 MHz, CD_3_OD, 20 °C) of 2*R*,3*R*,4*S*-catechin-4β-sulfonic acid (**8**). Figure S1: ^1^H NMR of sulfited catechin **2**, Figure S2: ^13^C NMR of sulfited catechin **2**, Figure S3: ^1^H NMR of sulfited epicatechin **4**, Figure S4: ^13^C NMR of sulfited epicatechin **4**, Figure S5: ^1^H NMR of C-4 sulfited catechin **8**.
